# Induction of a Cellular DNA Damage Response by Porcine Circovirus Type 2 Facilitates Viral Replication and Mediates Apoptotic Responses

**DOI:** 10.1038/srep39444

**Published:** 2016-12-16

**Authors:** Li Wei, Shanshan Zhu, Jing Wang, Rong Quan, Xu Yan, Zixue Li, Lei Hou, Naidong Wang, Yi Yang, Haijun Jiang, Jue Liu

**Affiliations:** 1 Beijing Key Laboratory for Prevention and Control of Infectious Diseases in Livestock and Poultry, Institute of Animal Husbandry and Veterinary Medicine, Beijing Academy of Agriculture and Forestry Sciences, No. 9 Shuguang Garden Middle Road, Haidian District, Beijing 100097, China; 2 Laboratory of Functional Proteomics and Research Center of Reverse Vaccinology, College of Veterinary Medicine, Hunan Agricultural University, Furong District, Changsha 410128, China

## Abstract

Cellular DNA damage response (DDR) triggered by infection of DNA viruses mediate cell cycle checkpoint activation, DNA repair, or apoptosis induction. In the present study, infection of porcine circovirus type 2 (PCV2), which serves as a major etiological agent of PCV2-associated diseases (PCVAD), was found to elicit a DNA damage response (DDR) as observed by the phosphorylation of H2AX and RPA32 following infection. The response requires active viral replication, and all the ATM (ataxia telangiectasia-mutated kinase), ATR (ATM- and Rad3-related kinase), and DNA-PK (DNA-dependent protein kinase) are the transducers of the DDR signaling events in the PCV2-infected cells as demonstrated by the phosphorylation of ATM, ATR, and DNA-PK signalings as well as reductions in their activations after treatment with specific kinase inhibitors. Inhibitions of ATM, ATR, and DNA-PK activations block viral replication and prevent apoptotic responses as observed by decreases in cleaved poly-ADP ribose polymerase (PARP) and caspase-3 as well as fragmented DNA following PCV2 infection. These results reveal that PCV2 is able to exploit the cellular DNA damage response machinery for its own efficient replication and for apoptosis induction, further extending our understanding for the molecular mechanism of PCV2 infection.

Porcine circovirus type 2 (PCV2), serving as a member of the family Circoviridae^1^, has been demonstrated to associate with postweaning multisystemic wasting syndrome (PMWS) and other clinical diseases, including porcine reproductive failure, dermatitis and nephropathy syndrome, necrotizing tracheitis, fetal myocarditis as well as congenital tremors, which is collectively considered as PCV2-associated diseases (PCVAD)^2^. In general, severely PCV2-infected pigs may disrupt immune system and develop immunosuppression, leading to an enhanced susceptibility to other etiological agents and a lowered immune response to vaccinations^2^. PCVAD is now endemic in many swine-rearing regions, and increasingly recognized as a serious threat to the swine industry worldwide^2^.

Five major open reading frames (ORFs) have been now identified in PCV2-infected cells. ORF1, a *rep* gene, encodes a viral replication-associated protein^3^; and ORF2, a *cap* gene, encodes a capsid protein which serves as a major immunogen for host-protection^4^. Besides the ORF1 and ORF2 proteins, ORF3 and ORF4 proteins are considered to participate in viral pathogenesis via apoptotic and anti-apoptotic functions following PCV2 infection, respectively^5^,^6^; ORF5 protein has been shown to involve in activation of NF-κB and prolonging of cell cycle S-phase^7^.

Cellular DNA damage induced by intrinsic or extrinsic insults activates a DNA damage response (DDR) that produces a complex protein kinase signaling cascade including cell cycle checkpoint activation, DNA repair, or apoptosis induction^8^. Following DNA damage, cellular conserved DDR pathways were rapidly activated^9^,^10^. These DDR pathways are involved in three related phosphatidylinositol 3-kinase-like kinases (PI3Ks): ataxia telangiectasia-mutated kinase (ATM), ATM-Rad3-related kinase (ATR), and DNA-dependent protein kinase (DNA-PK)^11^,^12^,^13^. ATM primarily responds to the presence of DNA double-strand breaks (DSBs) and is recruited and activated by the cellular MRN complex, which consists of the Mre11, Rad50, and Nbs1 proteins. ATR is mainly stimulated as a result of single-stranded DNA breaks and stalled DNA replication forks^9^,^14^, while DNA-PK responds to DSBs and involves DNA repair via the non-homologous end joining pathway (NHEJ)^15^,^16^. The DNA-PK holoenzyme is composed of the catalytic subunit of DNA-PK (DNA-PKcs) and two regulatory subunits, Ku70 and Ku86 heterodimer. Ku70/Ku86 heterodimer directly recognizes DSBs and mediates DNA-PKcs^17^. Investigation of downstream signalings shows that ATR predominantly phosphorylates Chk1, while ATM activates Chk2 phosphorylation^18^. Chk2 is also a substrate of ATR and DNA-PK^19^,^20^. In addition, ATM, ATR, and DNA-PK have all been demonstrated to activate p53 phosphorylation. Once at the damage site, these DDR kinases phosphorylate amounts of substrates including RPA32, H2AX, Chk1, Chk2, Nbs1, and p53 that followed by targeting other proteins, whereby leading to cell cycle arrest or induction of apoptosis^9^,^21^,^22^.

Infection of DNA viruses has been shown to induce a cellular DNA damage response, which can prevent or facilitate viral DNA replication, and promote the damaged DNA repair, cell cycle checkpoint activation or apoptotic responses in infected cells^23^,^24^. For adenovirus, the DDR constitutes an obstacle that must be surmounted for viral replication^25^,^26^. In contrast, some other viruses, including polyomavirus, simian virus type 40 (SV40), parvovirus minute virus of mice (MVM), herpes simplex virus type 1 (HSV-1), human cytomegalovirus, human papillomavirus (HPV), and MVC-bocavirus, trigger a DDR that facilitates their replication or a fully permissive infection^27^,^28^,^29^,^30^,^31^,^32^,^33^. As a DNA virus, there is still no report on a DDR induced by PCV2 infection and the DDR contributes to PCV2 replication and apoptotic responses.

In the present study, we have shown that PCV2 infection triggers a DDR as evidenced by phosphorylation of H2AX, RPA32, Nbs1, Chk1, Chk2 and p53 in the cultured cells. We further showed that all ATM-, ATR-, and DNA-PK-mediated pathways are involved in the PCV2 infection-induced DDR, which facilitates for PCV2 replication and PCV2-mediated apoptotic cell death.

## Results

### PCV2 infection induces a DDR in infected cells

**Figure 1:**
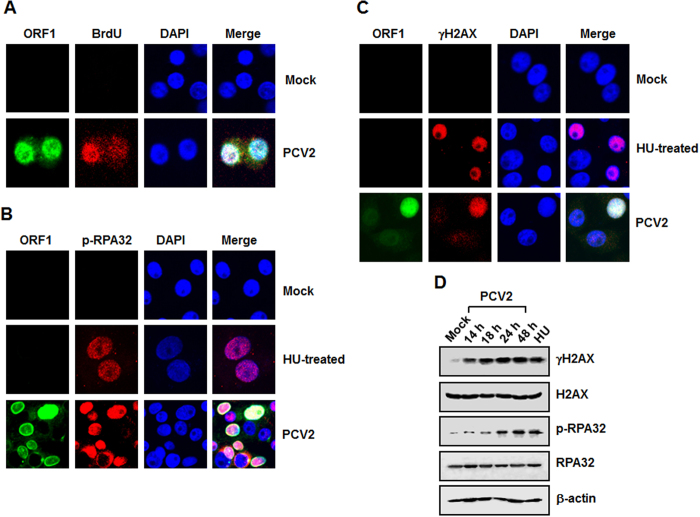
PCV2 infection induces a DNA damage response. (**A**) Utilization of BrdU incorporation for detection of the PCV2 DNA replication centers. Punctate staining by anti-ORF1 (green) or anti-BrdU (red) antibody indicates the viral replication centers. (**B** and **C**) Immunofluorescence assay of PCV2 infection-induced DDR. At 48 h postinfection, the PCV2-infected cells were coimmunostained with anti-PCV2 ORF1 (green) and anti-p-RPA32 (Ser33) (Red) (**B**) or anti-PCV2 ORF1 (green) and anti-γH2AX (green) (**C**). Cells treated with hydroxyurea (HU) were acted as a positive control for induction of DDR. Nuclei were visualized by DAPI staining. (**D**) Western blot analysis of PCV2 infection-induced DDR. The PCV2-infected cells were collected at the indicated times postinfection, and the whole-cell lysates were prepared and subjected to SDS-PAGE followed by immunoblotting. Phosphorylation of the DDR markers RPA32 and H2AX was assessed using the relevant antibodies. HU-treated cells served as a positive control, and β-actin was acted as a loading control of protein extracts. p-, phosphorylated.

To examine whether a DDR is activated during PCV2 infection, we evaluated the phosphorylation levels of DDR substrates H2AX and RPA32 in the PCV2-infected cells. First, we exploited BrdU incorporation to identify the PCV2 DNA replication centers and examined whether anti-BrdU staining colocalizes with the PCV2 ORF1 protein, which is responsible for PCV2 replication^3^. As shown in Fig. 1A, ORF1 protein was present at the active replication foci, which was stained by anti-BrdU. In subsequent experiments, we utilized anti-ORF1 staining as an indicator for the PCV2 DNA replication centers.

We continued to coimmunostain the PCV2-infected cells with anti-ORF1 and anti-phosphorylated H2AX at serine 139 (γH2AX), or with anti-ORF1 and anti-phosphorylated RPA32 at serine 33 (p-RPA32) antibodies, followed by staining with DAPI. In parallel, hydroxyurea (HU), a known inducer of the DDR, was used as a positive control. At 48 h postinfection, we found that PCV2 infection caused significant increases in the levels of γH2AX and p-RPA32 in ORF1-expressing cells (Fig. 1B and C, PCV2-infected), with most of the ORF1-expressing cells (green) also positive for anti-γH2AX and p-RPA32 (red), respectively. In the HU-treated cells, expressions of γH2AX and p-RPA32 were also present in the nuclei (Fig. 1B and C). We further determined the amounts of colocalized cells and found that there were 19.6% of cells positive for PCV2 viral antigens and all of them exhibited phosphorylation of H2AX and RPA32. Thus, PCV2 infection specifically leads to H2AX and RPA32 phosphorylation.

We also used Western blotting to examine the phosphorylation levels of H2AX and RPA32 in the PCV2-infected cells and found that they were increasingly phosphorylated over time (Fig. 1D). Incubation with PBS was used as mock-infected controls. The amounts of phosphorylated H2AX and RPA32 were evident at 14 h, the maximal activations were seen at 48 h postinfection (Fig. 1D). The increased levels of H2AX and RPA32 phosphorylation were concurrent with the expression of viral protein in the PCV2-infected cells (data not shown). In contrast, the total amounts of H2AX and RPA32 proteins did not substantially change during PCV2 infection as compared to those in the mock-infected cells. These results demonstrated that PCV2 infection induces a significant DDR in the cultured PK15 cells.

### ATM, ATR, and DNA-PK are all activated in the PCV2 infection-induced DDR

**Figure 2:**
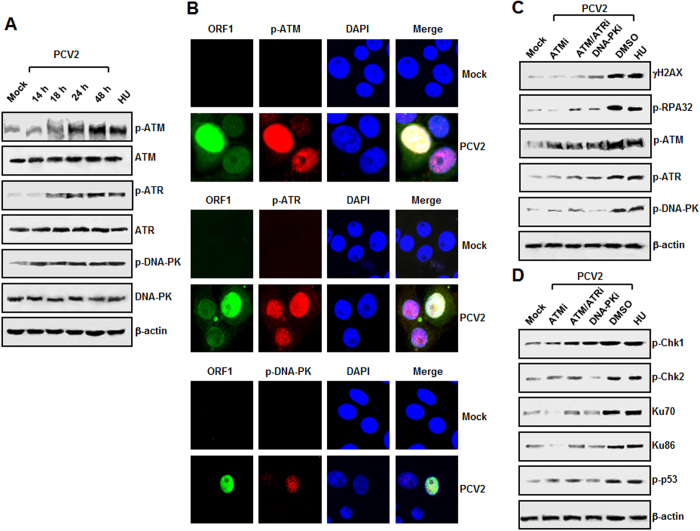
PCV2 infection-induced DDR involves ATM, ATR, and DNA-PK pathways. (**A**) PCV2-infected cells were collected at the indicated time points postinfection, and whole-cell lysates were prepared and subjected to SDS-PAGE followed by immunoblotting. The protein levels of phosphorylated ATM, ATR, and DNA-PK were analyzed. (**B**) Immunofluorescence analysis of PCV2 infection-induced ATM, ATR, and DNA-PK activations. At 48 h postinfection, the PCV2-infected cells were coimmunostained with anti-PCV2 ORF1 (green) and anti-p-ATM (Ser1981) (Red), anti-PCV2 ORF1 (green) and anti-p-ATR (Ser 428) (red), or anti-PCV2 ORF1 (green) and anti-p-DNA-PK (Thr2609) (red). Nuclei were visualized by DAPI staining. (**C**) Treatment with the specific inhibitors reduces PCV2-induced phosphorylation of ATM, ATR, and DNA-PK. PK15 cells were inoculated with PCV2 in the presence or absence of the specific inhibitor for ATM kinase (ATMi), ATM/ATR kinase (ATM/ATRi), or DNA-PK kinase (DNA-PKi). Cell lysates at 48 h postinfection were harvested and subjected to SDS-PAGE followed by immunoblotting with antibodies to ATM, ATR, and DNA-PK phosphorylated forms. (**D**) Treatment with the specific inhibitors reduces PCV2-induced phosphorylation of several DDR downstream targets. PK15 cells were inoculated with PCV2 in the presence or absence of the DDR kinase inhibitors. Cell lysates at 48 h postinfection were harvested and subjected to SDS-PAGE followed by immunoblotting with antibodies to Chk1, Chk2, and p53 phosphorylated forms as well as Ku80 and Ku70. β-actin was used as the loading control. p-, phosphorylated.

In order to address which kinase pathway might take responsible for the DDR induced during PCV2 infection. First, we used Western blotting to examine the degrees of ATM, ATR, and DNA-PK phosphorylation in the PCV2-infected cells. As shown in Fig. 2A, PCV2 infection led to progressive accumulations of ATM, ATR, and DNA-PK signals over time, the maximal inductions were seen at 48 h postinfection. In contrast, the total amounts of ATM, ATR, and DNA-PK proteins remained unchanged at various time points after PCV2 infection as compared to those in the mock-infected cells. As a positive control, HU treatment has been included in the experiment.

We continued to coimmunostain the PCV2-infected cells with anti-ORF1 and anti-phosphorylated ATM (p-ATM), anti-ORF1 and anti-phosphorylated ATR (p-ATR), or anti-ORF1 and anti-phosphorylated DNA-PK (p-DNA-PK) antibodies, followed by staining with DAPI at 48 h postinfection. As shown in Fig. 2B, PCV2 infection caused significant increases in the phosphorylation levels of ATM, ATR, and DNA-PK in the ORF1-expressing cells, with most of the ORF1-expressing cells (green) also positive for anti-p-ATM, -ATR, and -DNA-PK (red), respectively. We also found that all the positive cells (20.1%) for PCV2 viral antigens exhibited phosphorylation of ATM, ATR, or DNA-PK. Thus, PCV2 infection specifically induces the phosphorylation of ATM, ATR, and DNA-PK.

We then determined the effects of specific pharmacological inhibitors to ATM, ATR, and DNA-PK on the phosphorylation of these kinases. Trypan blue exclusion staining was used to test for cell viability and the indicated concentrations of these inhibitors exhibited no toxic effect on the inhibitors-treated cells (data not shown). We treated the PCV2-infected cells with the specific inhibitors at the indicated concentrations and determined the phosphorylation levels of H2AX, RPA32, ATM, ATR, and DNA-PK by Western blotting. As shown in Fig. 2C, all the three inhibitors decreased phosphorylated H2AX, RPA32, ATM, ATR, and DNA-PK levels as compared with those in DMSO-treated PCV2-infected cells. In addition, not only did these inhibitors decrease phosphorylation of target kinases but of non-target ones as well (Fig. 2C).

We also examined downstream DNA damage effector kinases after PCV2 infection and observed phosphorylation of Chk1 (Ser345) (Fig. 2D), in response to ATR activation^34^; phosphorylation of Chk2 (Thr68) and p53 (Ser15) (Fig. 2D), downstream targets of ATM/ATR^35^,^36^; and expression of Ku70 and Ku86 proteins (Fig. 2D), two regulatory subunits of the DNA-PK holoenzyme. After treatment with these specific inhibitors targeting ATM, ATR, and DNA-PK signals, decreased Chk1, Chk2, p53 phosphorylation, as well as reductions in Ku70 and Ku86 expressions were observed in the PCV2-infected cells regardless of the indicated inhibitor (Fig. 2D), as compared with those in DMSO-treated PCV2-infected cells.

Taken together, these results indicated that the PCV2 infection-induced DDR involves activation of ATM, ATR, and DNA-PK signaling pathways. Furthermore, an indirect inhibition of ATM, ATR, or DNA-PK signaling was also observed when the PCV2-infected cells were treated with non-targeting kinase inhibitor, which might be associated with reduction of PCV2 replication after inhibition of their kinase activation. Therefore, these data also implicated that phosphorylation of these three kinase signalings as a result of PCV2 infection led to the activation of downstream substrates.

### PCV2-induced DDR requires viral replication

**Figure 3:**
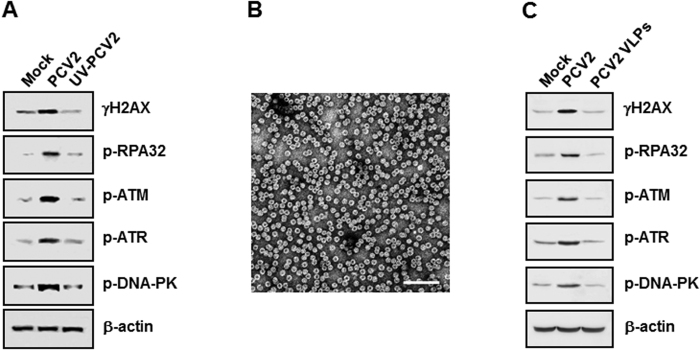
PCV2-induced DDR requires viral replication. **(A)** PK15 monolayer cells were infected with wild-type or UV-irradiated PCV2 at an MOI of 1 TCID_50_. (**B**) Images of PCV2 VLPs. Bar, 100 nm. (**C**) PK15 monolayer cells were inoculated with PCV2 virus-like particles (10 VLPs per cell). Cell lysates at 48 h post infection or inoculation were harvested and subjected to SDS-PAGE followed by immunoblotting with antibodies to H2AX, RPA32, ATM, ATR, and DNA-PK phosphorylated forms. β-actin was acted as the loading control. p-, phosphorylated.

To examine whether PCV2 replication was essential for DDR signalings, we used Western blotting to evaluate the phosphorylation levels of H2AX, RPA32, ATM, ATR, and DNA-PK in the cultured cells when inoculated with an UV-irradiated virus sample (corresponding to a MOI of 1 TCID_50_). PK15 cells were infected an UV-unirradiated or -irradiated virus samples for 48 h postinfection. The complete elimination of viral infectivity by UV irradiation was confirmed as determined by assaying virus titer with UV-irradiated undiluted viral suspension (data not shown). As expected, a significant increase in the phosphorylation levels of H2AX, RPA32, ATM, ATR, or DNA-PK was seen in the UV-unirradiated PCV2-infected cells when compared to those in the mock-infected cells (Fig. 3A). In contrast, the phosphorylation levels of H2AX, RPA32, ATM, ATR, or DNA-PK fell to its marginal level in the UV-irradiated PCV2-infected cells at 48 h postinfection as seen in the mock-infected cells (Fig. 3A). We also used PCV2 virus-like particles (VLPs)^37^ (Fig. 3B) to inoculate into PK15 monolayer cells and collected the 48 h PCV2 VLP-inoculated lysates for determination of the DDR activation. Inoculation of PCV2 VLP can only induce the marginal phosphorylation levels of H2AX, RPA32, ATM, ATR, or DNA-PK in the PK15 cultured cells (Fig. 3C), comparable to those in the mock-inoculated cells. Overall, these results demonstrated that PCV2 active replication was associated with induction of the cellular DNA damage response.

### The MRN complex is activated after PCV2 infection

**Figure 4:**
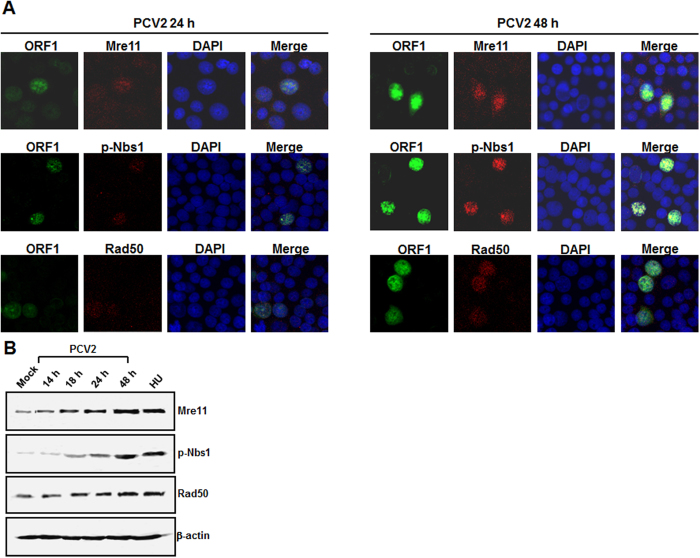
The MRN complex is activated after PCV2 infection. (**A**) Immunofluorescence observation of localization of the MRN complex to the PCV2 replication center. At 24 and 48 h postinfection, Mre11, p-Nbs1, and Rad50 were determined for colocalization with PCV2 ORF1 with their respective antibodies. Nuclei were visualized by DAPI staining. (**B**) Western blotting of PCV2-infected cells. The PCV2-infected cells were collected at the indicated times postinfection and were analyzed by immunoblotting for the levels of Mre11, p-Nbs1, and Rad50 by use of their respective antibodies. β-actin was acted as the loading control.

The MRN complex, which is composed of Mre11, Nbs1, and Rad50, has been considered to facilitate ATM and ATR activation^38^. To assess the activation of the MRN complex, PK15 cells were infected with PCV2 strain BJW at an MOI of 1 TCID_50_. We first sought relocalization of the MRN complex to the damage site within the nucleus, which is essential for a functional response^39^. Indirect immunofluorescence assay (IFA) was exploited to determine localization of the MRN complex at 24 and 48 h postinfection. At 24 h postinfection, Mre11, Nbs1, and Rad50 colocalized with the replication foci (punctuate patterns) in the PCV2-infected cells (Fig. 4A); in mock-infected cells, Mre11, Nbs1, and Rad50 were widely present in the nuclei without exhibiting any bright foci (data not shown). At 48 h postinfection, all three MRN proteins showed substantial increase levels in these locations (Fig. 4A). We also found that all the PCV2-positive cells (9.5% and 20.6% for 24 and 28 h postinfection, respectively) exhibited phosphorylation of Nbs1 and nuclear accumulation of Mre11 and Rad50. We then used Western blotting to assess the expression levels of the MRN complex. In the PCV2-infected cells, all three proteins Mre11, Nbs1, and Rad50 remained elevated over time, the maximal activations were seen at 48 h postinfection (Fig. 4B). These results from the localization of the MRN complex to the PCV2 replication foci indicate that as DNA damage, PCV2 genomes are sensed by the MRN complex.

### The DDR pathways are required for PCV2 infection

**Figure 5:**
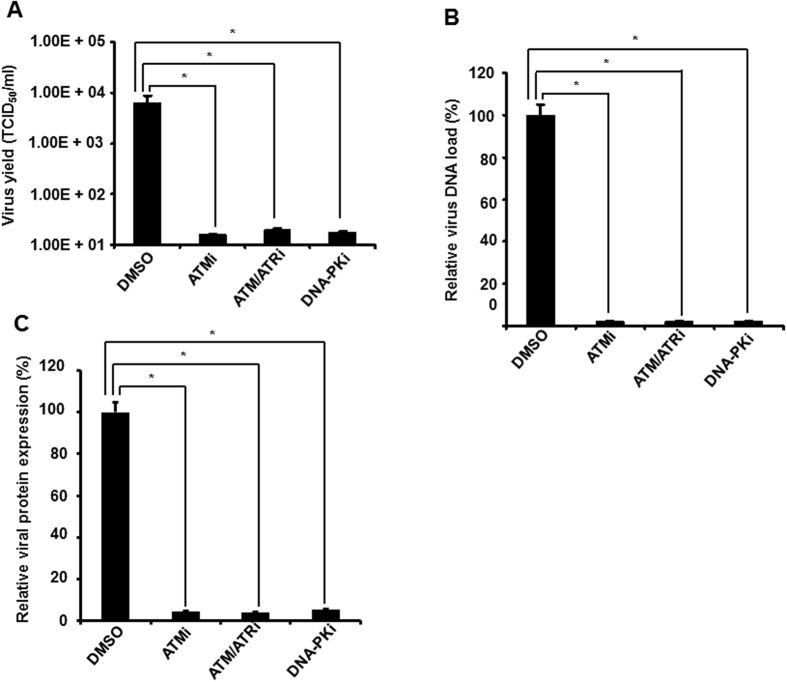
Activation of DDR is essential for efficient PCV2 growth. (**A**) Inhibition of DDR activation blocks PCV2 yield production. PCV2-infected PK15 cells 48 h after treatment with ATM kinase inhibitor (ATMi), ATM/ATR kinase inhibitor (ATM/ATRi), or DNA-PK inhibitor (DNA-PKi) were inoculated into PK15 monolayer cells and virus production was assayed by IFA under a microscope. Virus titers were expressed as TCID_50_ per milliliter and values are means ± SD of the results of three independent experiments. (**B**) Effect of DDR inactivation on PCV2 DNA replication. Supernatants of the PCV2-infected PK15 cells were performed by real-time PCR analysis after 48 h of treatment with these three inhibitors for amounts of PCV2 DNA load. Data for the PCV2-infected cells treated with the inhibitor are percentages of the value for the PCV2-infected untreated cells (means ± SD of values from three independent experiments). (**C**) Effect of DDR inactivation on PCV2 protein expression. The PCV2-infected PK15 cells were assayed by IFA after 48 h of treatment with these three inhibitors for amounts of PCV2 viral ORF1 synthesis. The amounts of PCV2 ORF1 protein expression are expressed as percentages of the ORF1-expressing signals in the PCV2-infected untreated cells. Data are means ± SD from three independent experiments. *P < 0.05 for a comparison of the PCV2-infected and PCV2-infected inhibitor-treated cells.

To assess whether PCV2 replication was impaired by blockage of any of the ATM, ATR, and DNA-PK pathways, we determined the effect of these kinase signalings on virus production in the PCV2-infected cells when treated with these kinase inhibitors. PK15 cells were infected with PCV2 in the absence of presence of specific pharmacological inhibitor to ATM, ATR, or DNA-PK phosphorylation and determined the virus titers present in the PCV2-infected cell culture supernatant at 48 h postinfection by using an IFA assay. As shown in Fig. 5A, ATM, ATM/ATR, or DNA-PK inhibitor treatment reduced progeny virus production 2.6-, 2.5-, or 2.55-log, respectively, indicating that DDR inactivation reduced PCV2 production.

To discern the mechanism underlying the reduced viral yield upon DDR inactivation, we further determined the effect of these DDR inhibitors on different stages of the PCV2 life cycle in the PK15 cultured cells. The PK15 cells were cultured in the presence or absence of the DDR inhibitors and infected with PCV2 strain BJW for 48 h. The cell culture supernatants were collected for real-time PCR to calculate DNA replication. As shown in Fig. 5B, treatment of cells with ATM, ATM/ATR, or DNA-PK inhibitor decreased the level of PCV2 viral DNA approximately 46.6-, 41.6-, or 42.5-fold, respectively. Concurrently, the ORF1 protein expression of the PCV2-infected cells in the presence or absence of these inhibitors was analyzed by IFA. The ORF1 protein expressions were significantly decreased when treated with these three inhibitors, as evidenced by the enhanced number of PCV2-positive cells seen in the infected cells (Fig. 5C). No significant differences were observed in the ORF1 protein expression between DMSO-treated PCV2-infected cells and untreated PCV2-infected cells (data not shown).

These results of reductions in PCV2 DNA replication and viral protein expression followed by decreased production of infectious PCV2 particles in cells after DDR inactivation suggest that PCV2 is capable of exploiting the cellular DDR machinery for its productive replication.

### Disruption of DDR activation prevents PCV2-mediated apoptotic responses

**Figure 6:**
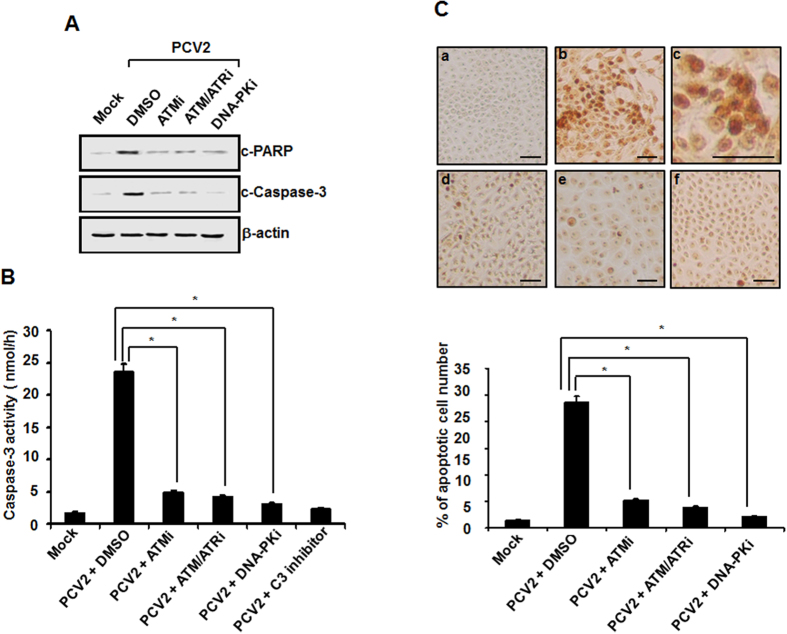
Inactivation of PCV2 stimulation of the DDR pathway suppresses virus-mediated apoptotic responses in host cells. (**A**) Monolayers of PK15 cells were infected with PCV2 in the presence or absence of the DDR inhibitors. At 48 h postinfection, the cell lysates were collected for Western blot analysis with specific antibodies to detect the cleavage of PARP and caspase-3. Equal protein loads were verified with β-actin blots. p-, phosphorylated; c, cleaved. (**B**) Whole-cell lysates collected from the PCV2-infected cells at 48 h after treatment with the DDR inhibitors were assayed for DEVDase activity by using the caspase-3 colorimetric DEVD-AFC. Furthermore, the cells infected with PCV2 alone were treated with DEVD-CHO, a caspase-3 inhibitor. Mock-infected cells were acted as a negative control. Values expressed are means from three independent experiments. (**C**) Inhibition of DDR activation reduces apoptosis caused by PCV2 infection by TUNEL staining. The PCV2-infected cells at 48 h after treatment with the DDR inhibitors were processed for the TUNEL assay. Upper panel, shown is TUNEL staining for fragmented DNA. The cells were mock infected (a), and infected with PCV2 in the absence (b,c is partial enlargement of b) or the presence of ATM (d), ATM/ATR (e), or DNA-PK inhibitor (f). Brown dots are TUNEL-positive nuclei. Bars, 80 μM. Data are representative of data from an experiment performed in repeated three times with similar results. Lower panel, TUNEL positivity was determined as the average percentages of apoptotic nuclei. Data are means ± SD from three independent experiments. *P < 0.05 for a comparison of the PCV2-infected and PCV2-infected DDR inhibitor-treated cells.

Research data have shown that the DNA damage kinases activate cell cycle checkpoint kinases^40^ thereby casing cell cycle arrest or induction of apoptosis^9^,^41^. PCV2 infection has been shown to mediate apoptotic cell death through activating caspase-8/3 pathway^5^. To evaluate the contribution of the DDR singalings to apoptosis induced by viral infection, we examined the effects of specific inhibitors of the DDR kinases on PCV2-induced apoptosis in the cultured cells. Firstly, we used Western blotting to measure the cleavage of host proteins related to characteristic hallmark features of apoptotic responses, including poly-ADP ribose polymerase (PARP) and caspase-3. PK15 cells were infected with PCV2 at an MOI of 1 TCID_50_ for 48 h in the presence or absence of ATM, ATM/ATR, or DNA-PK inhibitor at the indicated doses. Cell lysates were then collected and subjected to Western blotting. As shown in Fig. 6A, PCV2 infection led to the cleavage of PARP and caspase-3 at 48 h postinfection. When the DDR pathways were blocked by treatment of these three inhibitors, amounts of cleaved PARP and caspase-3 were significantly reduced. Secondarily, we used spectrofluorometric assay of proteolytic activity to measure caspase-3 activity in the PCV2-infected cells at 48 h postinfection after treatment with these three inhibitors. As expected, infection of PCV2 alone activated caspase-3 in the cultured cells, whereas their activities were significantly decreased in the infected cells when treated with the ATM, ATM/ATR, or DNA-PK inhibitor (Fig. 6B). A basal caspase-3 activity was only detected in the mock-infected cells. Furthermore, an internal control, a peptide inhibitor (Ac-DEVD-CHO) of caspase-3 activity, was used to confirm assay validity. Finally, we used a TUNEL assay to monitor apoptotic cell death at the cellular level. A brown signal was regarded as a TUNEL-positive cell. As shown in Fig. 6C, there was a significant reduction in TUNEL-positive cells (5.23%, 3.87%, or 2.15%) in the PCV2-infected cells at 48 h after treatment of the ATM, ATM/ATR, or DNA-PK inhibitor, respectively, as compared to the 23.56% TUNEL positivity observed in the PCV2-infected untreated cells. Only 1.8% positivity at 48 h postinfection was observed in the mock-infected cells. Overall, these results suggested that the DDR signaling pathways participate in the regulation of PCV2-induced apoptotic responses.

## Discussion

In this study, we investigated the interaction of PCV2 with the cellular DNA damage response pathway. Here, we demonstrated that PCV2 replication is capable of mediating DNA damage responses including ATM, ATR, and DNA-PK pathways, characterized by the phosphorylation of H2AX, RPA32, Nbs1, Chk1, Chk2, ATM, ATR, and DNA-PK. The majority of these phosphoproteins accumulated at the PCV2-induced replication compartment within the nuclei of the cultured cells. Inhibition of the DDR pathways induced by PCV2 infection leads to a decrease of viral activity, as demonstrated by reductions in viral DNA replication, virus protein expression, and virus production. We further showed that PCV2 infection-mediated apoptotic responses, as determined by increases of cleaved PARP and caspase-3 as well as fragmentary DNA by TUNEL staining, were blocked in the cultured cells, when activation of the DDR pathway was inhibited. These results indicated that infection with PCV2 leads to activation of the DDR signalings, playing key roles in the virus infection and virus infection-mediated apoptotic responses.

Research data have increasingly shown that varieties of viruses are able to induce activation of DDR in host cells. HSV-1, SV40, HPV, MVM, and human cytomegalovirus have all been shown to predominantly induce the ATM-mediated DDR following infections, whereby amounts of repair factors are triggered and recruited to the replication centers^27^,^29^,^32^,^33^,^42^. ATR activation and subsequent RPA32 phosphorylation have been demonstrated to play an important role in the DDR pathway induced by infection with Epstein-Barr virus (EBV), HIV, and adenoviruses^43^,^44^. In the case of parvoviruses, coinfection of AAV2 adenovirus promotes an ATM-, ATR-, and DNA-PK-mediated DDR^45^,^46^, but activation of the DDR signaling in response to AAV2 replication appears to predominantly induce the DNA-PK kinase^45^,^46^. In the present study, we demonstrated that infection of PCV2 stimulates DDR activation including all ATM, ATR, and DNA-PK signalings in the cultured cells, as evidenced by increased accumulations of ATM, ATR, and DNA-PK phosphorylations as well as their downstream effectors H2AX and RPA32 phosphorylations (Figs 1D and 2A), and colocalization of these kinase phosplorylations with PCV2 ORF1 protein in the viral replication centers (Figs 1B,C and 2B) after infection. Activation of the ATM, ATR, and DNA-PK-mediated DDR by PCV2 infection has been further confirmed by the data that a number of DDR kinase factors including Chk1, Chk2, and p53 phosphorylation, or regulatory subunits Ku70 and Ku86 expression (Fig. 2C and D) were reduced in the PCV2-infected cells after DDR inactivation by treatment with specific inhibitors. In addition, the induction of DDR requires viral replication (Fig. 3A and C), as observed for many other viruses^27^,^29^,^32^,^33^,^42^,^44^. This is in contrast to that infection with UV-inactivated AAV2 was capable of inducing an ATM/ATR-mediated DDR^47^,^48^, however, activation of the DNA-PK DDR requires AAV2 DNA replication^45^,^46^. Overall, these results indicate that the cellular response to PCV2 infection may be affected by several DDR regulators, including ATM, ATR, and DNA-PK-mediated DDR signalings.

Accumulating evidence has shown that a number of viral factors are likely associated with virus infections-mediated DDR activation. For the polyomaviruses, ATM-mediated DDR upon polyomavirus SV40 infection might be due to the large T antigen via Bul1 binding^49^. HPV genome replication appears to change from theta to rolling circle replication^50^, which may activate ATM signaling. AAV2 Rep78 elicits a DDR by introducing nonspecific nicks in the cellular DNA^51^, but it served as a minor contributor to the AA2 infection-induced DDR^46^. ATM-mediated DDR activation by parvovirus H-1 was reported to be associated with viral NS1-induced reactive oxygen species (ROS)^52^. The DNA replication of MVC genome was shown to take responsible for MVC-mediated DDR activation rather than the viral proteins^27^,^53^. As a single-stranded DNA virus, PCV2 genome replicates by a rolling-circle replication form, which might help to elicit strong DDR activation, as observed for HPV and MVC genome replication^27^,^50^,^53^. In addition, we observed accumulations of phosphorylated ATM, ATR, and DNA-PK in the PCV2 replication centers (Fig. 2B), it is possible that these kinases and ORF1 protein interact at these sites. It has been shown that ATM, ATR, or DNA-PK are capable of phosphorylating several proteins on well characterized (S/T)QE or (S/T)Q sites^54^. By alignment, we found that one conservative SQ motif is present in PCV2 ORF1 protein (data not shown), implicating that the viral replicase ORF1 protein might be associated with PCV2-induced DDR activation. However, the mechanism underlying PCV2 infection-induced DDR signaling needs to be further studied in the future.

DSBs serves as a senor for ATM activation, the MRN complex participates in its initial processing and plays a critical role in ATM-mediated DDR^55^. The MRN complex was essential for inducing DDR induction during HSV-1 infection^30^ and HPV infection^32^. In addition, the destruction of the MRN complex such as a loss of Mre11 has been reported following AAV2^56^, HSV-1^57^, MVM^27^, and MVC-bocavirus^31^ infections. Similar to the case for HSV^30^ and AVV2^56^, we found the accumulation of the MRN constituents at sites of viral replication within the infected cell nuclei (Fig. 4A). As reported for human cytomegalovirus infection^58^, PCV2 does not degrade the MRN complex, since the total levels of all three proteins of the MRN complex proven to be elevated changes in their steady-state proteins profiles following infection (Fig. 4B). High levels of total Mre11, p-Nbs1, and Rad50 protein expression could be due to the robust sequestration of the whole complex into the viral replication compartments (Fig. 4A). It is possible that the relocalization of the MRN constituents to the damage site is required to effectively activate the MRN complex^39^, thereby promoting PCV2-mediated DDR.

The DDR, serves as an important cellular response, which senses and repairs damaged DNA, is essential for maintenance of genome integrity. For virus infection, DDR involves in the host cellular antiviral mechanism to benefit the elimination of the invaded viral genome, but many viruses are capable of exploiting the DDR mechanism to promote their own replication^43^. In this study, we found that inhibition of the ATM, ATR, and DNA-PK kinases led to reduced PCV2 yield production (Fig. 5A), which is related to reductions in viral DNA replication (Fig. 5B) and viral protein synthesis (Fig. 5C), thus suggesting that activation of the DDR pathway enhances viral replication and is adopted by the virus for its own productive infection. Similar observations have been obtained with MVM^27^, polyomavirus^28^, HSV-1^30^, MVC-bocavirus^31^, HPV 16^32^, papovaviruses SV40^33^, and baculoviruses^59^. It is not clear whether enhancement of PCV2 replication by activation of the DDR signalings is direct or indirect. Thus, whether ATM/ATR and/or DNA-PK phosphorylate Rep and whether this contributes to PCV2 replication requires further study.

Many viruses exploit activation of the DDR signalings as a strategy to trigger apoptotic response through a p53-dependent or -independent pathway during virus infection^41^. In the present study, we demonstrated that blockage of the DDR pathway with specific kinase inhibitors for ATM, ATR, and DNA-PK activation significantly reduced the cleavage levels of PARP and caspase-3 as well as decreased DNA fragmentation triggered by PCV2 infection (Fig. 6A to C). We also found that p53 was phosphorylated upon PCV2 infection (Fig. 2D). This might implicate in apoptosis mediated by DDR activation in a p53-dependent fashion. This is in agreement with the reports that showed a role of the DDR signaling in inducing apoptosis during infection with MVC-bocavirus^31^ and Baculoviruses^59^. Therefore, we have expanded the data showing that the DDR pathway has an apoptotic role by promoting the onset of premature virus-mediated PARP cleavage, caspase activation and DNA fragmentation during PCV2 infection.

In conclusion, the results presented here showed that activation of ATM, ATR, and DNA-PK-mediated DDR upon PCV2 infection required PCV2 replication and is involved in induction of apoptotic response in the cultured cells. Knowledge of the role of DDR activation in regulating viral replication and mediating apoptotic responses upon PCV2 infection will extend our understanding for the molecular mechanism of PCV2 pathogenesis.

## Methods

### Virus and cells

PK15 cells were grown in minimal essential medium (MEM), which was supplemented with 5% heat-inactivated fetal bovine serum (FBS), 5% L-glutamine, 100 U of penicillin G/ml, and 100 μl of streptomycin/ml, at 37 °C in a humidified 5% CO_2_ incubator. PCV2 strain BJW^5^ was inoculated onto monolayer PK15 cells at a multiplicity of infection (MOI) of one 50% tissue culture infective dose units (TCID_50_) per cell. Additionally, the cells were carried out with 300 mM D-glucosamine treatment at 18–24 h after inoculation.

### Reagents and antibodies

Hydroxyurea (HU) (Sigma) diluted in deionized water was utilized at a final 2 mM concentration. InSolution ATM kinase inhibitor, ATM/ATR kinase inhibitor, and DNA-PK inhibitor II were obtained from Calbiochem and used at a final concentration of 7.5, 5, and 10 μM diluted in dimethyl sulfoxide (DMSO). 5-Bromo-2′-deoxyuridine (BrdU) (Sigma) was diluted in deionized water as a stock solution of 5 mM concentration. The cytotoxicity of these reagents on PK15 cells was assayed by trypan blue exclusion dye staining, showing no detectable cell death to the PK15 cells at the indicated doses. Cells were pretreated with the indicated inhibitors for 60 min before PCV2 infection and for infection duration unless otherwise indicated.

Anti-phosphorylated H2AX (anti-γH2AX) (Ser139) (Santa Cruz), anti-phosphorylated RPA32 (anti-p-RAP32) (Ser33) (Epitomics), anti-p-ATM (Ser1981), p-DNA-PK (Thr 2609), anti-DNA-PK, anti-Ku70, and anti-Ku86 (Santa Cruz), and anti-H2AX, anti-RAP32, anti-ATM, and anti-ATR (Calbiochem) were used in the present study. Antibodies specific for Rad50, Mrel1, p-Nbs1 (Ser343), p53 (Ser15), p-ATR (Ser428), p-Chk1 (Ser345), and p-Chk2 (Thr68) were purchased from Cell Signaling Technology. Mouse monoclonal Anti-BrdU antibody was obtained from Sigma. Horseradish peroxidase (HRP)-linked secondary antibodies were obtained from Sigma. Fluorescein isothiocyanate (FITC)- and tetraethyl rhodamine isothiocyanate (TRITC)-conjugated secondary antibodies were obtained from DAKO.

### Indirect immunofluorescence assay (IFA) and confocal microscopy

PK15 monolayer cells grown in chamber slides (BD) were infected with PCV2 strain BJW. At the indicated times postinfection, the cultured cells were washed with phosphate-buffered saline (PBS) and then fixed in 4% paraformaldehyde (PFA) diluted in PBS. The cells were co-incubated with guinea pig anti-ORF1 antibody and relevant DDR signal antibodies diluted in 3% bovine serum albumin (BSA)-PBS at room temperature (RT) for 1 h followed by incubation with fluorescein isothiocyanate (FITC)- and rhodamine-conjugated antibodies (DAKO) at 37 °C for 1 h. Nuclei were stained with 4′,6′-diaminido-2-phenylindole (DAPI). The cells were then rinsed in dH_2_O, dried and mounted with fluorescence mounting media, and observed under Nikon AIR confocal laser microscope system. In addition, immunofluorescence images were quantitatively determined in over 100 cells on each of 3 occasions.

For BrdU incorporation, PK15 cells were grown on a chamber slide and inoculated with PCV2 at an MOI of 1 TCID_50_. At 18 h postinfection, BrdU was supplemented into the cell culture medium at a final concentration of 5 μM. At 24 h postinfection, cells were fixed and coimmunostained with guinea pig anti-PCV2 ORF1 and mouse anti-BrdU antibody to mark the PCV2 replication centers.

### Virus infectivity assay

PK15 monolayer cells were infected with PCV2 strain BJW at an MOI of 1 TCID_50_ in the presence of ATM, ATR, or DNA-PK inhibitors. At 48 h postinfection, the cell cultures were harvested by three cycles of freeze-thawing and subjected to clarification. Virus infectivity was determined by IFA as described previously^60^.

### Inoculation of PCV2 virus-like particles (VLPs)

PCV2 VLPs were prepared as described by Zhang *et al*.^37^ and inoculated into PK15 monolayer cells for 48 h for determination of DNA damage responses activation.

### Whole cell lysates

Whole cell lysates from the cultured PK15 cells after PCV2 infection or inoculation at the indicated time points were prepared by using the Nuclear Extract kit (Active Motif) in accordance with the manufacturer’s protocol.

### Western blotting

The whole cell lysates prepared were diluted in 2× sample buffer and boiled for 5 min. 20 μg of each extract were resolved on 10–12% sodium dodecyl sulphate-polyacrylamide gel electrophoresis (SDS-PAGE) and transferred to nitrocellulose membranes with a semidry transfer cell (Bio-Rad). The membranes were blocked at RT for 2 h in blocking buffer TBST (20 mM Tris-HCl [pH 7.4], 150 mM NaCl, 0.1% Tween-20) containing 5% skim milk powder to block nonspecific binding, and then reacted with primary antibodies raised against ATM, ATR, DNA-PK, p-ATM, p-ATR, p-DNA-PK, H2AX, RPA32, γH2AX, p-RPA32, Mrel1, p-Nbs1, Rad50, p-p53, p-Chk1, p-Chk2, Ku70, Ku86, as well as β-actin at RT for 2 h followed by incubation with HRP-conjugated secondary antibodies diluted in blocking TBST buffer at RT for 1 h. Immunoreactive bands were observed using enhanced chemiluminescence system (Kodak Image Station 4000R).

### TUNEL assay

PK15 monolayer cells were infected with PCV2 at an MOI of 1 TCID_50_ with or without treatment of ATM, ATR, or DNA-PK inhibitor. A DeadEnd colorimetric TUNEL (terminal deoxynucleotidyl transferase-mediated dUTP-biotin nick end labeling) system kit (Promega, Madison, WI) was used to detect apoptosis as described previously^60^.

### Fluorimetric assay of caspase-3 activity

A BD ApoAlert caspase fluorescent assay kit (Clontech) was adopted to determine caspase-3 activity of the PCV2-infected PK15 cells after ATM, ATR, or DNA-PK inhibitor treatment as described previously^60^.

### Statistical analysis

Results are presented as averages ± the standard deviations or standard errors of the means. Student’s *t* test is used to make statistical comparisons, and differences between groups are considered significant if the *P* value is <0.05.

## Additional Information

**How to cite this article**: Wei, L. *et al*. Induction of a Cellular DNA Damage Response by Porcine Circovirus Type 2 Facilitates Viral Replication and Mediates Apoptotic Responses. *Sci. Rep.* **6**, 39444; doi: 10.1038/srep39444 (2016).

**Publisher’s note:** Springer Nature remains neutral with regard to jurisdictional claims in published maps and institutional affiliations.
